# Pulmonary Administration of TLR2/6 Agonist after Allergic Sensitization Inhibits Airway Hyper-Responsiveness and Recruits Natural Killer Cells in Lung Parenchyma

**DOI:** 10.3390/ijms25179606

**Published:** 2024-09-04

**Authors:** Justine Devulder, Mathieu Barrier, Julie Carrard, Latiffa Amniai, Coline Plé, Philippe Marquillies, Valérie Ledroit, Bernhard Ryffel, Anne Tsicopoulos, Patricia de Nadai, Catherine Duez

**Affiliations:** 1 Univ. Lille, CNRS, Inserm, CHU Lille, Institut Pasteur de Lille, U1019-UMR 9017-CIIL—Center for Infection and Immunity of Lille, F-59000 Lille, France; j.devulder@imperial.ac.uk (J.D.); anne.tsicopoulos@pasteur-lille.fr (A.T.); patricia.de-nadai@pasteur-lille.fr (P.d.N.); 2 CNRS and University Orleans—INEM (Immuno-Neuro Modulation), UMR7355 INEM, 45071 Orleans , France; bernhard.ryffel@cnrs-orleans.fr

**Keywords:** allergic asthma, Toll-like receptor-2, natural killer cell

## Abstract

Asthma is a chronic lung disease with persistent airway inflammation, bronchial hyper-reactivity, mucus overproduction, and airway remodeling. Antagonizing T2 responses by triggering the immune system with microbial components such as Toll-like receptors (TLRs) has been suggested as a therapeutic concept for allergic asthma. The aim of this study was to evaluate the effect of a TLR2/6 agonist, FSL-1 (Pam2CGDPKHPKSF), administered by intranasal instillation after an allergic airway reaction was established in the ovalbumin (OVA) mouse model and to analyze the role of natural killer (NK) cells in this effect. We showed that FSL-1 decreased established OVA-induced airway hyper-responsiveness and eosinophilic inflammation but did not reduce the T2 or T17 response. FSL-1 increased the recruitment and activation of NK cells in the lung parenchyma and modified the repartition of NK cell subsets in lung compartments. Finally, the transfer or depletion of NK cells did not modify airway hyper-responsiveness and eosinophilia after OVA and/or FSL-1 treatment. Thus, the administration of FSL-1 reduces airway hyper-responsiveness and bronchoalveolar lavage eosinophilia. However, despite modifications of their functions following OVA sensitization, NK cells play no role in OVA-induced asthma and its inhibition by FSL-1. Therefore, the significance of NK cell functions and localization in the airways remains to be unraveled in asthma.

## 1. Introduction

Asthma is a chronic pulmonary disease characterized by persistent and non-resolving airway inflammation, bronchial hyper-reactivity, mucus overproduction, airway wall remodeling, and airway narrowing. Immunological studies have classified asthma into two main endotypes according to the immune responses: type 2 (T2)-high and T2-low. The allergic phenotype belongs to the T2-high endotype and is associated with the production of type 2 cytokines, such as interleukin (IL)-4, IL-5, and IL-13, and eosinophilic inflammation [1]. Allergic asthma is the most common asthma phenotype in the general asthma population, as allergic mechanisms are present in over 60% of asthmatics [2,3]. There is no cure for asthma, and current therapies, consisting of inhaled corticosteroids and bronchodilators, suppress symptoms rather than change the natural history of the disease. Additional asthma therapies, like biologicals, are used in severe asthma, defined as uncontrolled despite optimized maximal therapy [4]. Defining new therapeutic approaches for asthma is still an ongoing process. The idea of antagonizing T2 responses by triggering the immune system with microbial components has been suggested as therapeutic concept for allergic asthma. It is based on the statement that a loss of symbiotic relationships with a diverse range of beneficial microbes is responsible for the inadequate immune regulation involved in chronic immune diseases including allergic diseases [5,6,7,8].

Pattern recognition receptors (PRRs) are a group of innate conserved sensors expressed by a wide range of cells of the immune system and non-immune cells, such as epithelial cells, that recognize a wide range of microbial molecules. Among PRRs, Toll-like receptors (TLRs) are able to promote, exacerbate, or ameliorate airway inflammatory response in mouse models [9]. TLR2 forms heterodimers either with TLR1 to recognize triacylated lipopeptides or with TLR6 to recognize diacylated lipopeptides. These heterodimers are located at the cell membrane and recognize a large array of microbial compounds from bacteria, fungi, parasites, and viruses [10]. Polymorphisms in the *tlr2* and *tlr6* genes were found to be linked with the susceptibility to developing allergic asthma [11,12,13,14]. In animal models, TLR2 agonists induce different responses depending on the agonists, the dose, and the timing of the administration. In a murine model of chronic fungal asthma, *Tlr2*^−/−^ mice exhibited lower airway hyper-responsiveness, airway inflammation, and lung Th2 cytokine levels 7 days after the administration of *Aspergillus fumigatus* compared with the *Tlr2*^+/+^ group. However, 30 days after *A. fumigatus*, *Tlr2*^−/−^ mice showed enhanced airway neutrophil recruitment and airway hyper-responsiveness [15]. Consistent with these results, the sensitization of mice with ovalbumin (OVA) concomitantly with Pam3CSK4 (TLR1/2 agonist) aggravated bronchial hyper-reactivity, eosinophilic inflammation, and IL-5 and IL-13 concentration in the lungs [16], whereas intraperitoneal administration of Pam3CSK4 after each challenge of OVA reduced total cell and eosinophil numbers in the bronchoalveolar lavage fluid (BALF), pulmonary IL-4 and IL-5 levels, and airway hyper-responsiveness. This anti-inflammatory effect of Pam3CSK4 was independent of IL-10 and TGF-β but was dependent on IL-12p40, implicating a shift from a Th2 to a Th1 immune response [17]. Similarly, in a model of allergic airway inflammation induced by Timothy grass pollen extract, the administration of MALP-2 (TLR2/6 agonist) during the allergen challenge reduced eosinophilic inflammation and IL-5 and IL-10 production by lymph nodes [18]. Intratracheal treatment with macrophage-activating lipopeptide (MALP)-2 in combination with Th1-cytokine interferon (IFN)-γ after sensitization with OVA but before intranasal challenge reduced airway hyper-responsiveness, eosinophilia, and Th2 cytokines in the bronchoalveolar lavage fluid [19]. Finally, *Tlr6^−^*^/−^ mice treated with *Aspergillus fumigatus* and house dust mite (HDM) antigen exhibited increased airway hyper-responsiveness, inflammation, and remodeling compared with WT asthmatic groups, suggesting that TLR6 may exert protective effects in asthma [20].

Natural killer (NK) cells are innate lymphocytes that are critical for host protection against pathogens [21]. They express several TLRs, and TLR ligands can activate NK cells directly or indirectly [22,23,24,25,26]. For instance, the stimulation of purified human NK cells with *Mycobacterium bovis* leads to their activation directly through TLR2 [27]. In mice, spleen NK cells are directly activated by vaccinia virus through a TLR2-dependent mechanism [28]. Although the lung is one of the major reservoirs of NK cells in the body, where they represent about 10–20% of lymphocytes [29,30], their functions in asthma are still unclear [31]. In the blood and lung compartments, NK cells from asthma patients exhibit phenotypic and functional changes compared to healthy donors [32,33,34,35,36,37,38]. In mouse models of asthma, conflicting results on the role of NK cells have been obtained depending on the protocol and the tools used: NK cells either have pro-inflammatory properties, have no effect, or promote the resolution or suppression of allergic inflammation (reviewed in [31]).

The aim of the present study was to evaluate the effect of TLR2/6 agonist administered locally by nasal instillation after the allergic airway reaction was established in a mouse model to mimic potential treatment of allergic asthma. Mice were sensitized and challenged with OVA and then treated intranasally with the synthetic diacylated lipoprotein FSL-1 (Pam2CGDPKHPKSF). Here, we report that FSL-1 decreased established OVA-induced airway hyper-responsiveness (AHR) and eosinophilic inflammation and led to the recruitment of NK cells in lung tissue. However, FSL-1-activated NK cells were not involved in AHR and eosinophilia modification in the OVA asthma model.

## 2. Results

### 2.1. Pulmonary Administration of FSL-1 Decreases Established Ovalbumin-Induced Airway Hyper-Responsiveness and Eosinophilic Inflammation

**Figure 1:**
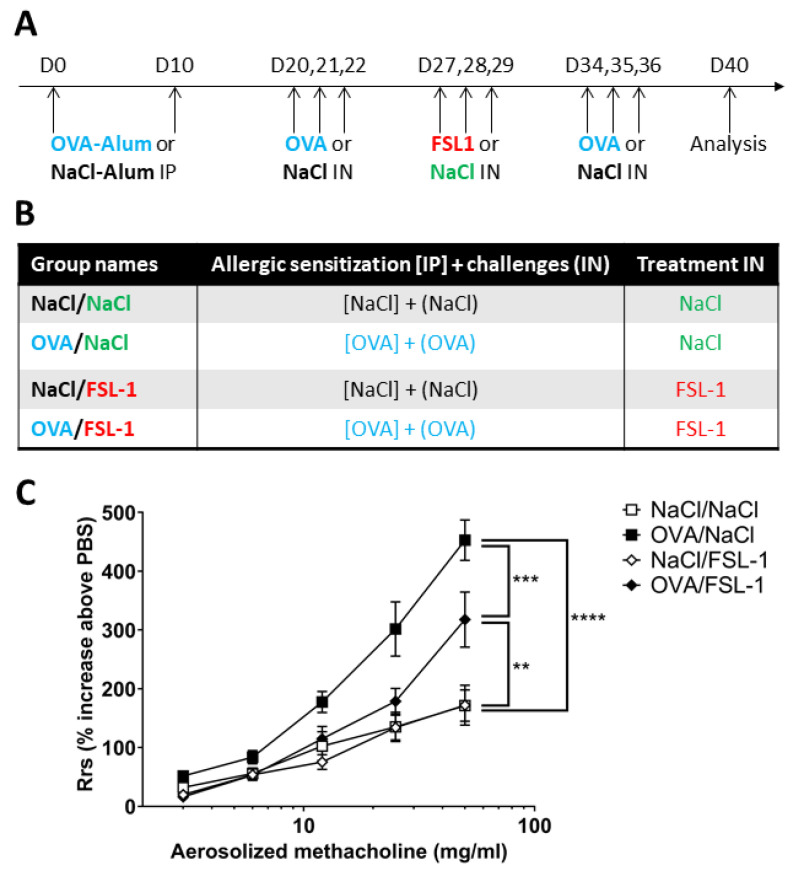
FSL-1 decreases airway hyper-responsiveness in OVA-sensitized female C57BL/6 mice. (**A**) Protocol for mouse sensitization and treatment. Mice were sensitized by intraperitoneal (IP) injection of 20 µg ovalbumin (OVA) emulsified in 100 µL of aluminum hydroxide on days 0 and 10. On days 20, 21, 22, 34, 35, and 36, mice were anesthetized with isoflurane and challenged by nasal instillation (IN) of 100 µg OVA in 25 µL saline solution (NaCl). Control group received intraperitoneal injection of NaCl emulsified in 100 µL of aluminum hydroxide and was challenged by nasal instillation of NaCl. FSL-1 treatment was administered on days 27, 28, and 29 by nasal instillation (0.5 µg/25 µL) on isoflurane-anesthetized mice. Mice were analyzed 96 h later on D40. (**B**) Description of groups of mice sensitized to OVA and controls (NaCl) treated with FSL-1 or control (NaCl). (**C**) Total respiratory resistance (Rrs). Results are expressed as percentage of Rrs increase for each methacholine dose in comparison to PBS. *n* = 10 NaCl/NaCl, *n* = 10 OVA/NaCl, *n* = 6 NaCl/FSL-1, *n* = 8 OVA/FSL-1. Statistical significance is shown for the highest methacholine dose: ** *p* < 0.01, *** *p* < 0.001, **** *p* < 0.0001 (two-way ANOVA followed by Tukey’s multiple comparisons test).

Ovalbumin (OVA)-sensitized mice receiving NaCl nasal instillation treatment (OVA/NaCl mice) exhibited significantly increased airway resistance (Rrs) compared to control NaCl/NaCl mice (Figure 1). Mice sensitized to ovalbumin and treated with FSL-1 (OVA/FSL-1) exhibited significant decreases in airway resistance, whereas FSL-1 did not modify airway resistance in control mice (NaCl/FSL-1). All mice showed similar baseline airway resistance (NaCl/NaCl: 0.907 ± 0.063, NaCl/FSL-1: 0.842 ± 0.044, OVA/FSL-1: 0.919 ± 0.040, and OVA/NaCl: 0.849 ± 0.052 cmH_2_O.s/mL).

**Figure 2:**
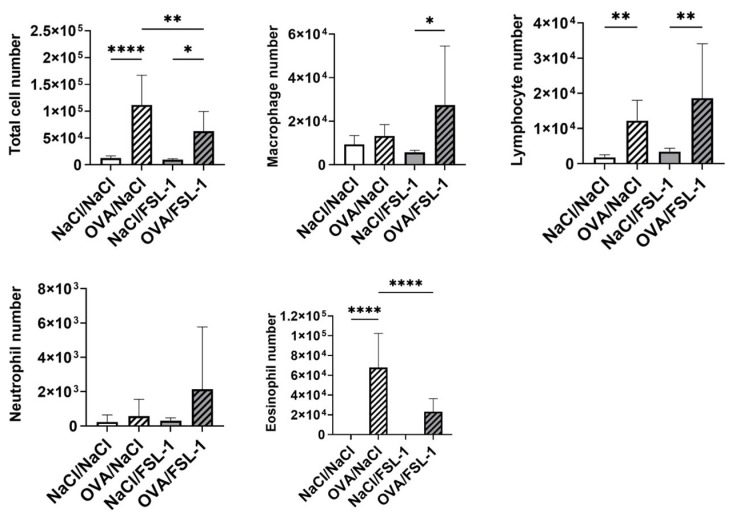
FSL-1 decreases bronchoalveolar lavage eosinophilia in OVA-sensitized female C57BL/6 mice. Results are expressed as number of cells. *n* = 9 NaCl/NaCl, *n* = 9 OVA/NaCl, *n* = 6 NaCl/FSL-1, *n* = 8 OVA/FSL-1. * *p* < 0.05, ** *p* < 0.01, **** *p* < 0.0001 (Shapiro–Wilk test for normality and one-way ANOVA followed by Tukey’s multiple comparisons test).

Total cell numbers in BAL from OVA-sensitized mice significantly increased in NaCl- or FSL-1-treated mice (Figure 2). In particular, lymphocyte and eosinophil numbers significantly increased in BAL from OVA-sensitized mice. Total cell and eosinophil numbers significantly decreased after nasal instillation of FSL-1.

**Figure 3:**
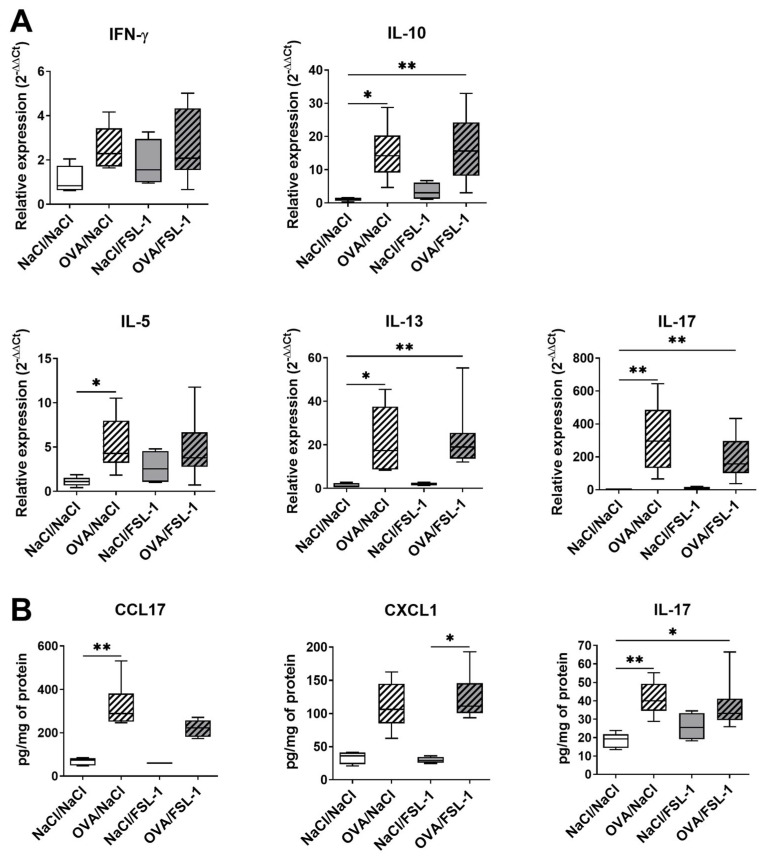
FSL-1 does not modify cytokine and chemokine expression in the lungs of OVA-sensitized female C57BL/6 mice. (**A**) mRNA expression in lung extracts by qRT-PCR. Relative expression was determined as 2^−ΔΔCt^. (**B**) Cytokine levels in lungs expressed as pg/mg of total lung proteins. *n* = 5 NaCl/NaCl, *n* = 6 OVA/NaCl, *n* = 4 NaCl/FSL-1, *n* = 8 OVA/FSL-1. * *p* < 0.05, ** *p* < 0.01 (Kruskal–Wallis test followed by Dunn’s multiple comparisons post-test).

In contrast, FSL-1 treatment did not affect the increased OVA-specific serum IgE induced by OVA sensitization (Figure S1). Regarding cytokine and chemokine expression and production in the lungs, OVA sensitization increased IFN-γ, IL-5, IL-13, IL-17, IL-10, CCL17, and CXCL1 levels, whether or not mice were treated with FSL-1 (Figure 3). FSL-1 treatment did not have any effect on cytokine and chemokine expression and production in mice sensitized to OVA or not. Airway hyper-responsiveness (AHR) and BAL eosinophilia were unaffected by FSL-1 treatment in TLR2-deficient mice, thus confirming the effect of FSL-1 was dependent upon TLR2 (Figure S2).

In conclusion, the TLR2/TLR6 agonist FSL-1 administered in the lungs by nasal instillation after the establishment of experimental asthma significantly decreased AHR and eosinophilia. FSL-1 treatment in the lungs did not modify the production of Th1-, Th2-, or Th17-type cytokines and associated chemokines in the lungs. As natural killer (NK) cells were described to express and to be directly activated through TLR2 [23,24,25,26,27], we next focused on this cell type.

### 2.2. NK Cells Are Recruited and Activated in the Lung after FSL-1 Treatment

**Figure 4:**
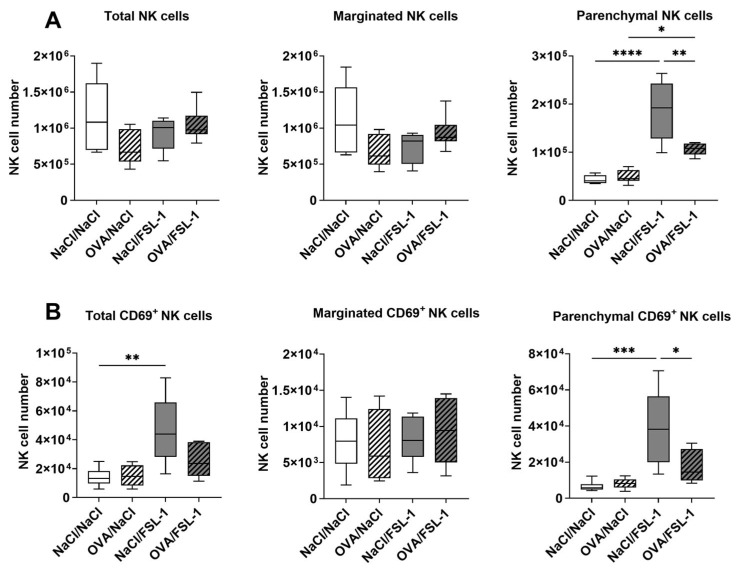
FSL-1 induced the recruitment and activation of NK cells in the parenchyma of NaCl treated, but not OVA-sensitized, female C57BL/6 mice. (**A**) The number of NK cells CD3^−^NK1.1^+^NKp46^+^ in the lungs, marginated or parenchymal, was analyzed by flow cytometry. (**B**) The number of activated NK cells CD3^−^NK1.1^+^NKp46^+^CD69^+^ was analyzed by flow cytometry. Results are expressed as number of cells. *n* = 6 NaCl/NaCl, *n* = 6 OVA/NaCl, *n* = 6 NaCl/FSL-1, *n* = 6 OVA/FSL-1. * *p* < 0.05, ** *p* < 0.01, *** *p* < 0.001, **** *p* < 0.0001 (Shapiro–Wilk test for normality and one-way ANOVA followed by Sidak’s multiple comparisons test).

We measured the number of CD3^−^NK1.1^+^NKp46^+^ natural killer (NK) cells in the whole lung by flow cytometry (Figure 4). In naïve mice, NK cells were described to be essentially localized in the vascular marginated compartment [39]. We aimed to identify whether NK cell location may be affected by allergic airway sensitization and FSL-1 treatment. The intravenous injection of fluorescently labeled anti-CD45 antibody prior to the ex vivo staining of lung NK cells with another anti-CD45 antibody labeled with a different fluorochrome allowed for the distinction of intravascular but marginated cells (CD45 double-positive) and parenchymal cells (CD45 single-positive), i.e., cells that had migrated through the pulmonary endothelium [39]. Total numbers of NK cells in the lungs were unchanged by OVA sensitization and FSL-1 treatment (Figure 4A, left panel). On average, 88% of NK cells were double-positive for CD45, i.e., marginated in the lungs, and only the number of parenchymal NK cells was significantly affected by treatments. Intranasal FSL-1 administration significantly increased parenchymal NK cell numbers in NaCl control mice. The number of parenchymal NK cells was not modified by OVA sensitization. However, OVA-sensitized mice exhibited fewer parenchymal NK cell numbers after FSL-1 treatment compared to NaCl control mice (Figure 4A). CD69 is a protein associated with lymphocyte activation and tissue residency [40]. Approximately 1% of marginated NK cells were CD69^+^, whereas CD69^+^ NK cells accounted for around 15% of parenchymal NK cells in NaCl/NaCl control group. The number of CD69^+^ NK cells was significantly increased in the parenchymal compartment by FSL-1 treatment in the NaCl control mice, and not-significantly in the OVA-sensitized animals (Figure 4B). Nevertheless, if mice were sensitized and challenged with OVA prior to FSL-1 treatment, the number of CD69^+^ NK cells was significantly decreased in the parenchymal compartment compared to the NaCl/FSL-1 group. Overall, FSL-1 treatment appears to increase the recruitment and activation of NK cells into the lung parenchyma, more particularly in the NaCl group.

**Figure 5:**
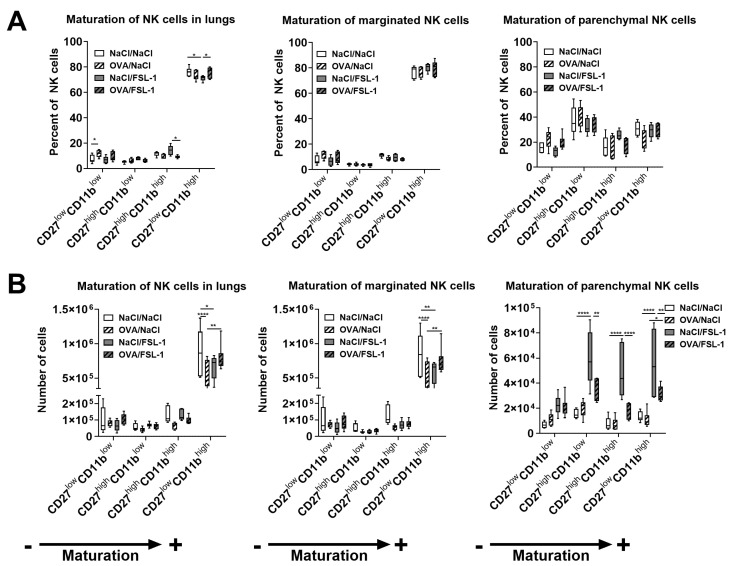
Repartition of NK cell subsets in lung compartments after FSL-1 treatment in OVA-sensitized or control female C57BL/6 mice. (**A**) Percentage of NK cell subsets defined by flow cytometry in the whole lungs and the marginated and the parenchymal compartment. (**B**) Total number of NK cell subsets in the whole lungs and the marginated and the parenchymal compartment. The direction of NK cell maturation (less mature to more mature NK cells) is indicated by the arrow and the signs + and − under the graphs. *n* = 6 NaCl/NaCl, *n* = 6 OVA/NaCl, *n* = 6 NaCl/FSL-1, *n* = 6 OVA/FSL-1. * *p* < 0.05, ** *p* < 0.01, **** *p* < 0.0001 (two-way ANOVA followed by Tukey’s multiple comparisons test).

We also analyzed the repartition of NK cell populations according to CD27 and CD11b expression, which reflects their maturation status; CD27^low^CD11b^low^ (double negative, DN) NK cells and CD27^low^CD11b^high^ NK cells are the least and the most mature, respectively [41]. OVA sensitization modified the repartition of NK cell populations in the whole lungs by significantly increasing the percentage of DN NK cells at the expense of the more mature CD27^high^CD11b^high^ (double-positive, DP) and CD27^low^CD11b^high^ NK cells. FSL-1 treatment of NaCl control mice significantly decreased the percentage of CD27^low^CD11b^high^ NK cells, while not-significantly increasing CD27^high^CD11b^low^ and DP NK cells. However, FSL-1 treatment of OVA-sensitized mice did not modify the repartition of NK cell subsets compared to OVA/NaCl mice (Figure 5A, left panel). When results were expressed as total cell number, it appeared that the most mature population of CD27^low^CD11b^high^ NK cells significantly decreased in OVA-sensitized animals and that FSL-1 treatment of OVA-sensitized mice significantly restored CD27^low^CD11b^high^ NK cell numbers. FSL-1 treatment of NaCl control mice significantly decreased CD27^low^CD11b^high^ NK cell numbers (Figure 5B, left panel). The same modifications were observed in the number of marginated intravascular NK cells (Figure 5A,B, middle panels). The repartition of NK cell subsets according to their maturation status was completely different in the parenchymal compartment compared to the whole lung. In control NaCl/NaCl animals, DN NK cells represented around 15%, CD27^high^CD11b^low^ NK cells around 35%, DP NK cells around 15%, and CD27^low^CD11b^high^ NK cells around 35% of parenchymal NK cells. No significant difference was observed in the percentage of the subsets depending on sensitization or treatment (Figure 5A, right panel). OVA sensitization did not modify the numbers of NK cell subsets in parenchyma. However, FSL-1 treatment increased the recruitment of all subsets to the parenchyma. The increase was significant for NaCl/FSL-1 mice (except for DN NK cells) and non-significant for OVA/FSL-1 mice (except for the CD27^low^CD11b^high^ NK cells). Nevertheless, if mice were sensitized and challenged with OVA prior to FSL-1 treatment, the increase in CD27^high^CD11b^low^, DP, and CD27^low^CD11b^high^ NK cell counts in the parenchyma was significantly diminished (Figure 5B, right panel). Altogether, these results indicate that FSL-1 administration in the lungs induced the recruitment of NK cells in the parenchyma, more particularly of the three more mature subsets, and that OVA sensitization partially prevented the recruitment of these subsets in the parenchyma.

**Figure 6:**
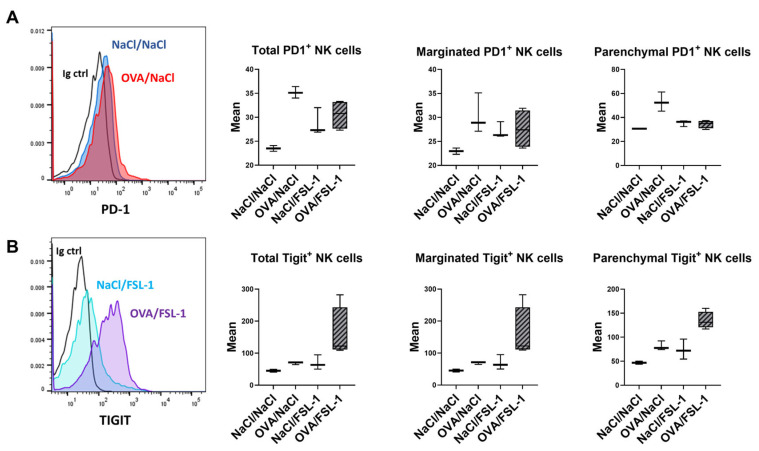
Expression of exhaustion markers on NK cells from mice sensitized with OVA with or without FSL-1 treatment. (**A**) Analysis of PD1 on NK cells in the whole lungs and in the marginated and parenchymal compartment by flow cytometry. (**B**) Expression of TIGIT on NK cells in the whole lungs and in the marginated or parenchymal compartment. Results are expressed as the mean fluorescence of exhaustion markers. *n* = 2 NaCl/NaCl, *n* = 3 OVA/NaCl, *n* = 3 NaCl/FSL-1, *n* = 4 OVA/FSL-1.

As OVA sensitization prior to FSL-1 treatment appears to prevent NK cell activation (CD69 expression) and the recruitment of the most mature NK cell subsets into the parenchyma, we measured the expression of a few checkpoint molecules known to decrease NK cell activation [42]. Parenchymal NK cells slightly increased the expression of the checkpoint molecule PD1 (Programmed cell death protein 1) only in OVA-sensitized mice (Figure 6A). FSL-1 treatment of OVA-sensitized mice increased TIGIT (T-cell immunoreceptor with immunoglobulin and immunoreceptor tyrosine-based inhibition motif domain) expression both in marginated intravascular and in parenchymal NK cells (Figure 6, right panel).

### 2.3. NK Cells Are Not Involved in Airway Hyper-Responsiveness and Eosinophilia Modifications Induced by OVA or FSL-1

**Figure 7:**
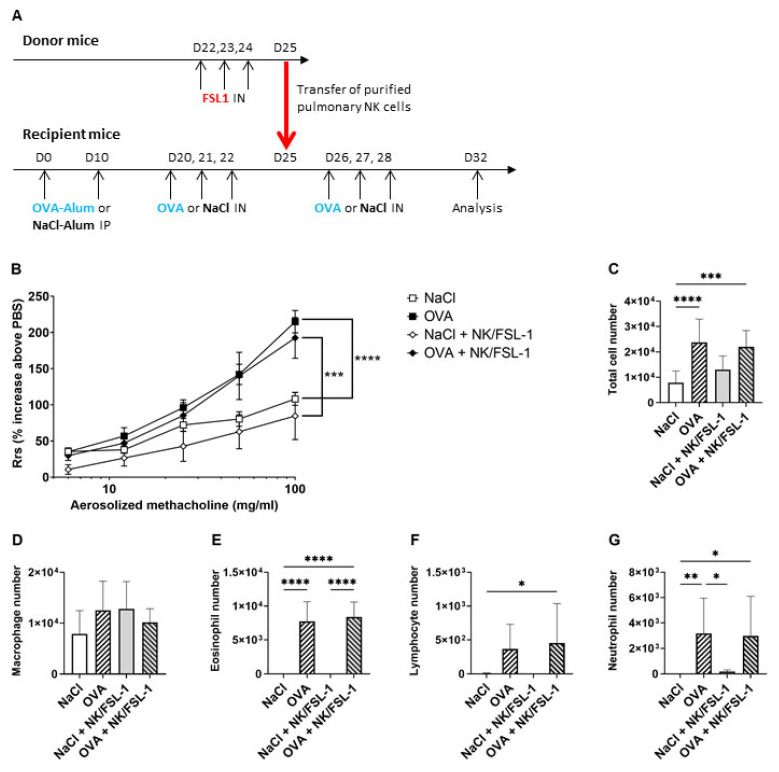
Transfer of lung NK cells isolated from FSL-1-treated mice modifies neither airway hyper-responsiveness nor bronchoalveolar lavage cellularity in OVA-sensitized female C57BL/6 mice. (**A**) Transfer protocol. Mice called “Donor mice” received nasal instillation of FSL-1 (0.5 µg/25 µL) after isoflurane-induced anesthesia on days 22, 23, and 24. On D25, lungs were collected and NK cells were purified by flow cytometry sorting. Recipient mice were sensitized with OVA as described in Figure 1A. On D25, they were transferred with 1 × 10^6^ purified NK cells in 50 µL, administered by intravenous injection under isoflurane-induced anesthesia. On days 26, 27, and 28, mice were anesthetized with isoflurane and challenged by nasal instillation (IN) of 100 µg OVA in 25 µL saline solution (NaCl) or NaCl for control group. Mice were analyzed 96 h later on D32. (**B**) Total respiratory resistance (Rrs). Results are expressed as percentage of Rrs increase for each methacholine dose in comparison to PBS. *n* = 9 NaCl, *n* = 15 OVA, *n* = 5 NaCl+NK/FSL-1, *n* = 9 OVA+NK/FSL-1, *** *p* < 0.001, **** *p* < 0.0001 (two-way ANOVA followed by Tukey’s multiple comparisons test). (**C**–**G**) Total cell, macrophage, eosinophil, lymphocyte, and neutrophil numbers in bronchoalveolar lavage. *n* = 9 NaCl, *n* = 15 OVA, *n* = 5 NaCl+NK/FSL-1, *n* = 9 OVA+NK/FSL-1. * *p* < 0.05, ** *p* < 0.01, *** *p* < 0.001, **** *p* < 0.0001 (Shapiro–Wilk test for normality and one-way ANOVA followed by Tukey’s multiple comparisons test).

The modifications of NK cell populations in the lungs of FSL-1-treated OVA-sensitized mice prompted us to evaluate their participation in the inhibitory effect of FSL-1. We used two different approaches. First, the lungs of FSL-1-treated mice were collected to sort NK cells with flow cytometry. The transfer of NK cells isolated from the whole lungs of FSL-1-treated mice into OVA-sensitized mice inhibited neither AHR (Figure 7A) nor BAL cellularity (Figure 7B–F). Therefore, this transfer did not reproduce the FSL-1 treatment of OVA-sensitized mice, suggesting that NK cells may not be involved in FSL-1’s effect.

**Figure 8:**
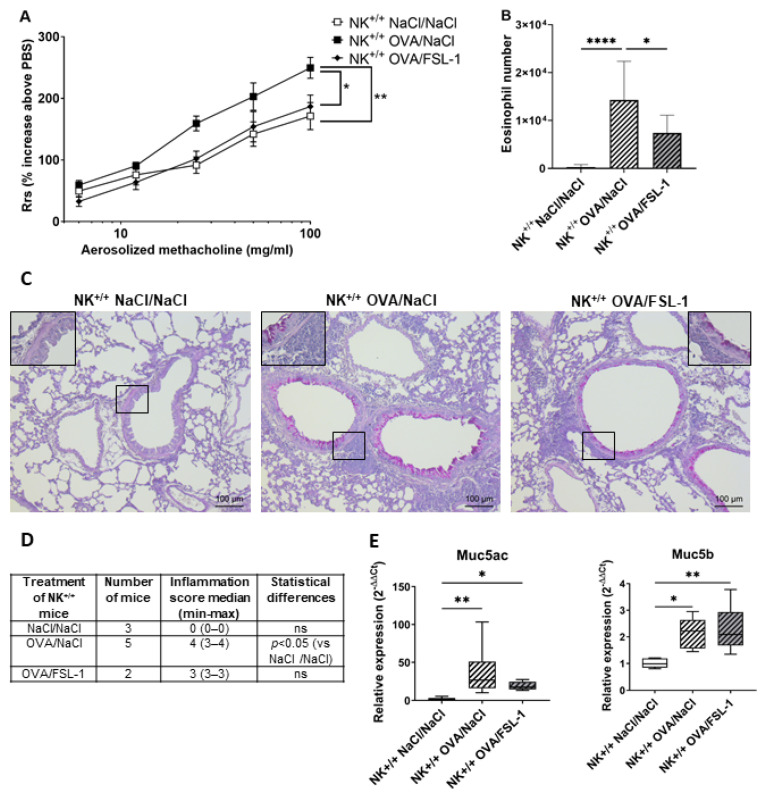
FSL-1 inhibits airway hyper-responsiveness and bronchoalveolar lavage eosinophilia in NK^+/+^ female mice but does not modify lung inflammation and mucus production. Airway resistance (**A**) and eosinophil numbers in bronchoalveolar lavage (**B**) were measured in littermate NK^+/+^ female mice treated as mentioned in Figure 1. NK^+/+^ mice: *n* = 9 NaCl/NaCl, *n* = 9 OVA/NaCl, *n* = 8 OVA/FSL-1. * *p* < 0.05, ** *p* < 0.01, **** *p* < 0.0001. (**A**) Statistical significance is shown for the highest methacholine dose (two-way ANOVA followed by Tukey’s multiple comparisons test). (**B**) Shapiro–Wilk test for normality, and one-way ANOVA followed by Tukey’s multiple comparisons test. (**C**) PAS staining was performed on lung sections of littermate NK^+/+^ mice either treated with NaCl/NaCl or OVA/NaCl, or OVA/FSL-1. For each group, one representative lung section is shown. (**D**) Semi-quantitative severity score (0–5) for inflammatory cell infiltration was determined for each mouse, 3 sections/mouse. Kruskal–Wallis test followed by Dunn’s multiple comparisons test. (**E**) mRNA expression of Muc5ac and Muc5b in lung extracts after qRT-PCR. Relative expression was determined as 2^−ΔΔCt^. HPRT was used as housekeeping gene. *n* = 5 NaCl/NaCl, *n* = 6 OVA/NaCl, *n* = 7 OVA/FSL-1. * *p* < 0.05, ** *p* < 0.01 (Kruskal–Wallis test followed by Dunn’s multiple comparisons post-test).

**Figure 9:**
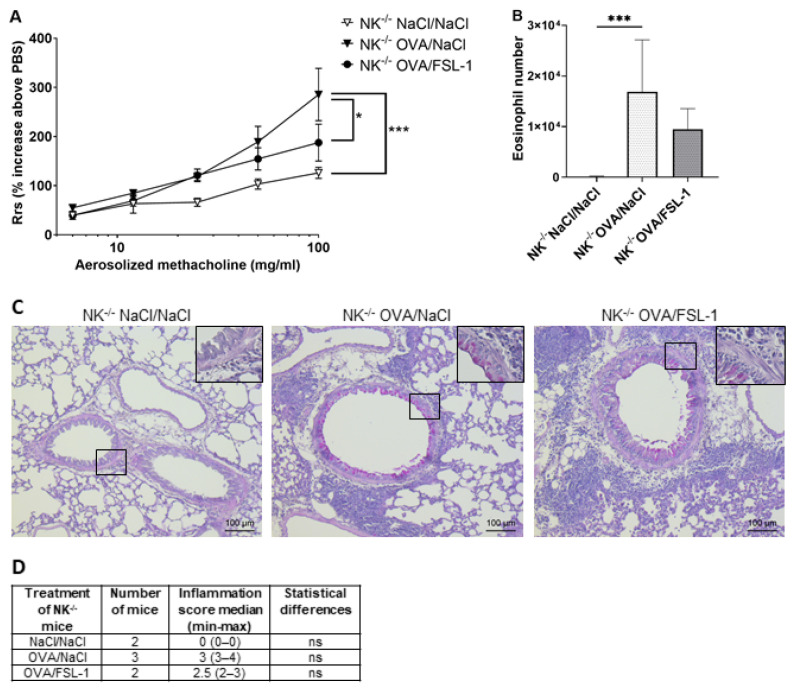
FSL-1-induced inhibition of airway hyper-responsiveness and bronchoalveolar lavage eosinophilia is independent of NK cells. Airway resistance (**A**) and eosinophil numbers in bronchoalveolar lavage (**B**) were measured in NK^−/−^ female mice treated as mentioned in Figure 1. NK^−/−^ mice: *n* = 6 NaCl/NaCl, *n* = 12 OVA/NaCl, *n* = 7 OVA/FSL-1. * *p* < 0.05, *** *p* < 0.001. (**A**) Statistical significance is shown for the highest methacholine dose (two-way ANOVA followed by Tukey’s multiple comparisons test). (**B**) Shapiro–Wilk test for normality and one-way ANOVA followed by Tukey’s multiple comparisons test. (**C**) PAS staining was performed on lung sections of NK^−/−^ mice either treated with NaCl/NaCl or OVA/NaCl or OVA/FSL-1. For each group, a representative lung section is shown. (**D**) Semi-quantitative severity score (0–5) for inflammatory cell infiltration was determined for each mouse, 3 sections/mouse. Kruskal–Wallis test followed by Dunn’s multiple comparisons test.

The second approach used NK cell-deficient mice obtained after crossing heterozygous Nkp46^iCre +/−^ mice with heterozygous R-DTA^+/−^ mice. We first checked that NK^+/+^ littermate mice (bred in-house) behaved like WT C57BL/6 mice (commercial mice). Treatment of OVA-sensitized mice with FSL-1 significantly decreased AHR, whether mice were females (Figure 8A,B and Figure S3) or males (Figure S4). BAL total cell numbers and eosinophilia was significantly inhibited in females (Figure 8B and Figure S3) but not in males (Figure S4). Periodic acid Shiff (PAS) staining on lung sections of NK^+/+^ OVA-sensitized mice showed no effect of FSL-1 treatment on inflammation and mucus production (Figure 8C,D), which paralleled lung Muc5ac and Muc5b expression (Figure 8E). Again, no effect of FLS-1 was seen on lung cytokine and chemokine production or expression (Figure S5). As previously demonstrated [43], female OVA-sensitized NK cell deficient mice developed AHR and increased BAL cellularity similar to their OVA-sensitized littermates (Figure S6). Likewise, male OVA-sensitized NK cell-deficient mice developed AHR and BAL cellularity similar to their OVA-sensitized littermates (Figure S7). These results confirmed that NK cells do not play a role in OVA-induced experimental asthma. Once these checks had been carried out, we analyzed the effect of FSL-1 treatment in OVA-sensitized NK^−/−^ mice. Similarly to littermate NK^+/+^ mice, FSL-1 treatment significantly decreased AHR and BAL inflammation in female NK^−/−^ mice (Figure 9A,B and Figures S8 and S9). The decrease in BAL eosinophilia was found to be close to significance (*p* = 0.0578). PAS staining revealed no differences in lung inflammation and mucus production between OVA-sensitized NK^−/−^ mice treated with FSL-1 or not (Figure 9C,D).

In conclusion, whereas NK cell proportions, activation, and migration toward the parenchyma were affected by FSL-1, NK cells played no role in FSL-1-induced inhibition of established OVA-induced experimental asthma.

## 3. Discussion

In this article, we aimed to evaluate whether pulmonary administration of TLR2/6 agonist FSL-1 to mice would be able to modify airway responses in an established allergic asthma model. We showed that both AHR and BAL eosinophilia were significantly decreased, but we did not measure differences in the production of Th1, Th2, nor Th17-type cytokines and associated chemokines in the lungs. Our results have some similarities with previously published data where TLR2/1 or TLR2/6 agonists were administered before or during challenges. Indeed, intraperitoneal administration of a TLR2/1 agonist or pulmonary (intratracheal and intranasal) administration of TLR2/6 agonists reduced Th2 responses and the associated allergic airway reactions in BALB/c mice [17,18,19]. The involved mechanisms included a shift from a Th2 to a Th1 immune response with the TLR2/1 agonist, as suggested by the increased IFN-γ and IL-10 but decreased IL-5 production after in vitro stimulation of lymph node cells and by the treatment of *Il-12*^−/−^ mice [17]. When BALB/c mice were subjected to a chronic exposure of Timothy grass pollen and treated with a TLR2/6 agonist once serum IgG1 levels raised, i.e., after sensitization but during challenges, the treatment attenuated BAL eosinophil counts, BAL IL-5, IL-4, CCL5 and IL-10, and lung remodeling. However, the reduction in the allergic phenotype was not caused by an increased frequency of CD4^+^/foxp3^+^ regulatory T cells or Th1 responses [18]. Finally, in OVA-sensitized BALB/c mice, TLR2/6 intratracheal administration before OVA challenge significantly decreased BAL IL-5 and IL-13 but increased BAL eosinophilia [19]. These studies used a different mouse strain (BALB/c instead of C57BL/6 mice) and a different allergen/sensitization protocol and TLR2 agonist. Nevertheless, the mechanism involved in the inhibitory effect of TLR2 agonist was unclear. As we did not evaluate cytokine production in lymph nodes, we cannot rule out an effect on T cell response. However, the particularity of our study is that FSL-1 was intranasally administered once the allergic reaction was established in the airways. We previously showed that this protocol was sufficient to induce AHR and airway inflammation [44]. We used subsequent OVA challenges in order to mimic allergen challenge in asthmatic allergic patients after FSL-1 treatment. Our results were robust and reproducible, as FSL-1’s inhibitory effect on established allergic asthma was confirmed on different mouse batches, bred either in commercial or our animal facilities and both in males and females. Nevertheless, FSL-1 treatment of OVA-sensitized mice did not reduce BAL eosinophilia in male mice, suggesting sex-related difference in this response.

We aimed to better understand the underlying mechanisms of FSL-1’s effect. Since TLR2 agonists are able to activate NK cells [27,28], we evaluated the effect of FSL-1 treatment on lung NK cells. Interestingly, the distinction of intravascular but marginated cells and parenchymal cells demonstrated variations in NK cell numbers depending on lung compartments which could not be identified in the whole lung. As previously shown [44], OVA sensitization and challenge did not modify total NK cell numbers in the lung. Here, we showed that most NK cells in the lungs were intravascular and a small proportion was parenchymal, as previously suggested both in mice and humans [30,39]. OVA treatment did not modify this distribution. However, local FSL-1 treatment increased parenchymal NK cell numbers, suggesting a migration toward the parenchyma. FSL-1 also increased the proportion of CD69^+^ NK cells in the parenchyma. The expression of CD69 is a marker of recent activation, but it also identifies tissue-resident lymphocytes [30]. In mice, NK cell subsets can be identified based on their CD27 and CD11b expression, which reflects their maturation status [41]. CD27^low^CD11b^high^ NK cells have been described to be the predominant subset in mouse lungs [29]. Here, we showed that this most mature population is the predominant subset among intravascular marginated cells, whereas a more balanced distribution was observed in the parenchymal compartment. OVA sensitization decreased the number of the most mature CD27^low^CD11b^high^ NK cells in the marginated compartment, whereas subsequent FSL-1 treatment significantly restored it. The role of marginated NK cells in asthma and allergic reactions is currently unknown. However, it was previously shown that marginated NK cells exhibit greater cytotoxicity than their circulating counterparts, have pro-inflammatory characteristics, and may play a critical role in eliminating aberrant circulating cells [45]. The equivalent distribution of NK cell subsets in the parenchyma was maintained after OVA and FSL-1 treatment. FSL-1 treatment increased the numbers of NK cells from the more mature subset in the parenchyma whether mice were OVA-sensitized or not. Interestingly, FSL-1 could not elicit the parenchymal recruitment of NK cells at the same level if mice were previously OVA-sensitized. This suggests that NK cells from “allergic asthmatic” mice were less responsive to FSL-1 treatment than those from naïve mice. This result is in accordance with the NK cell dysfunction observed in asthma patients [33]. Moreover, similarly to NK cells from severe asthma patients, which increase the expression of the TIM3 checkpoint molecule [33], lung NK cells from OVA-sensitized mice increased the expression of another checkpoint molecule (PD1). The level of expression of TIGIT was significantly increased after FSL-1 treatment in OVA-sensitized mice only. TIGIT is an inhibitory receptor which is up-regulated following the activation of both mouse and human NK cells and which limits NK cell responses [42,46]. We were not able to detect TIM3 expression with the commercially available antibodies.

Despite the modifications in NK cell populations in the lungs of FSL-1-treated OVA-sensitized mice, neither the transfer nor the depletion of NK cells modified airway responses (AHR and BAL eosinophilia) in FSL-1-treated OVA-sensitized mice, suggesting that NK cells did not play a role in this effect. We also confirmed that NK cells did not play a role in OVA-induced experimental asthma, as previously demonstrated [43].

In conclusion, FSL-1 delivered into the lungs was proven to be effective in reducing AHR and eosinophilia in a mouse model of allergic asthma once asthma was established. These results are in support of the existing literature on the suppression of asthma manifestations in mice [16,17,18,47,48,49]. However, one has to be careful as FSL-1 possesses adjuvant activity when administered jointly with an antigen and may induce Th2-type responses in vivo [50]. NK cell functions are abnormal in asthma, and we describe a modification in their localization in different lung compartments. However, they played no role in OVA-induced asthma and FSL-1 inhibition. Other conditions may be controlled by NK cell functions, as shown in a mouse model of asthma in the offspring of pollutant-exposed pregnant mice [51].

## 4. Materials and Methods

### 4.1. Mice

Wild-type (WT) C57BL/6JRj mice were obtained from Janvier Labs (France). *Nkp46*^iCre^ mice are knock-in mice in which the gene encoding the improved Cre (iCre) recombinase was inserted into the *Nkp46* locus [52]. They were obtained from Prof Eric Vivier (Centre d’Immunologie de Marseille-Luminy, Aix Marseille Université/INSERM/CNRS, France). Genetically modified mice expressing diphtheria toxin A under control of a loxP flanked stop cassette (R-DTA mice) [53] were obtained from Prof BN Lambrecht and Prof H Hammad (VIB-UGent Center for Inflammation Research, Belgium). Heterozygous *Nkp46*^iCre^ mice were crossed with heterozygous R-DTA mice. *Nkp46*^iCre +^ R-DTA^+^, *Nkp46*^iCre +^ R-DTA^-^, and *Nkp46*^iCre −^ R-DTA^+^ mice from these crossings were used as controls and called NK^+/+^ littermates, whereas *Nkp46*^iCre−^ R-DTA^−^ mice were NK cell-deficient and were called NK^−/−^ mice. Mice were housed and bred in Plateau Technique d’Experimentation et de Haute Technologie Animale (PLEHTA) of the Pasteur Institute in Lille in ventilated cages with clear and tinted plastic nest boxes, a controlled day/night cycle, and food and water ad libitum. We followed 3R rules, and all experimental procedures received authorization and complied with French law (n° 7874-2016070417344442v3). Seven- to ten-week-old mice maintained on an ovalbumin-free diet were used for the experiments.

### 4.2. Sensitization, Airway Challenges, and Treatment

Mice were sensitized with 20 μg of ovalbumin (OVA) (Grade V; Sigma-Aldrich, St. Louis, MO, USA), or NaCl 0.9% for control groups, emulsified in 2.25 mg aluminum hydroxide (Reheis, Berkeley Heights, NJ, USA) and challenged according to protocol described in Figure 1A. TLR2/TLR6 agonist treatment (synthetic diacylated lipoprotein FSL-1 [S-(2,3-bispalmitoyloxypropyl)-Cys-Gly-Asp-Pro-Lys-His-Pro-Lys-Ser-Phe,Pam2CGDPKHPKSF], InvivoGen, San Diego, CA, USA) or control NaCl was administrated by nasal instillation on isoflurane-anesthetized mice (0.5 µg/25 µL/mouse). All measurements were taken 96 h after the last OVA challenge. Group names are described in Figure 1B.

### 4.3. Lung Resistance Measurement

Lung resistance was assessed using the ventilator-based Flexi-Vent^®^ system (SciReq Inc., Montreal, QC, Canada). Mice were anesthetized (5 mL/kg body weight of 10% medetomidine (Pfizer, New York, NJ, USA) and 10% ketamine (Merial, Boehringer Ingelheim, Ingelheim, Germany) and immediately intubated with an 18-gauge catheter, followed by mechanical ventilation. As a support treatment for anesthesia, Pancuronium bromide (1%, 5 mL/kg, Organon, Jersey City, NJ, USA) was administered to WT C57BL/6JRj mice, and hyperventilation was performed for NK^+/+^ and NK^−/−^ mice as previously described [54]. Lung volume history was standardized with deep inflation maneuver (slow inflation from PEEP to 30 cm H_2_O with 3 s breath hold). Respiratory frequency was set at 150 breaths/min with a tidal volume of 0.2 mL, and a positive-end expiratory pressure of 2 mL H_2_O was applied. Mice were exposed to nebulized PBS followed by increasing concentrations of nebulized metacholine (0–100 mg/mL in PBS) (Sigma-Aldrich, St. Louis, MO, USA) using an ultrasonic nebulizer (Aeroneb, Aerogen, Galway, Ireland). For each dose, 10 cycles of nebulization and measurements were performed. Nebulization was performed during the first cycle and consisted of 20 puffs per 10 s, with each puff of aerosol delivery lasting 10 ms. For each cycle, measurements were obtained for 15 s followed by ventilation for 5 s. Baseline total respiratory resistance (Rrs) was restored before administration of the subsequent doses of methacholine. Only Rrs values corresponding to COD (Coefficient of determination) values > 0.95 were kept. For each dose, the maximum Rrs value measured was taken and was expressed as the percentage of maximum Rrs value measured after PBS exposure (% increase above PBS).

### 4.4. BAL Cellular Composition

Lungs were washed via the tracheal cannula with 1 mL of PBS. Total leucocyte numbers were counted, cytocentrifuged (Shandon cytospin 4; Thermo Fisher Scientific Inc., Waltham, MA, USA), and stained with May Grünwald Giemsa (Microm Microtech, Brignais, France). Cells were identified as macrophages, eosinophils, neutrophils, and lymphocytes by standard hematological procedures and at least 300 cells were counted under ×400 magnification.

### 4.5. Measurement of OVA-Specific IgE in Serum

Serum levels of OVA-specific IgE were measured by ELISA. Ninety-six-well plates (Corning, Avon, France) were coated with purified anti-IgE (2 µg/mL, clone R35-72, BD Biosciences). After overnight incubation (+4 °C) with serum dilutions, the binding of specific antibodies was detected by the addition of homemade biotinylated ovalbumin for specific IgE quantification. The binding of biotinylated proteins was revealed by addition of avidin–horseradish peroxidase (1/3000, Invitrogen, Carlsbad, CA, USA) and 3,3′,5,5′-tetramethylbenzidine (TMB) substrate solution (Interchim, Montluçon, France). The OVA-specific antibody titers of the samples were related to pooled standards generated in the laboratory. Results are expressed as the inverse of the dilution corresponding to 50% of the maximal OD.

### 4.6. Cytokine Measurements in Lung Homogenates

Lung protein extracts were prepared after mechanical dissociation (Precellys, Bertin Technologies SAS, Montigny-le-Bretonneux, France) of 3 right lobes in 1 mL of lysis buffer (Tissue-Protein Extraction Reagents, Life Technologies, Villebon-sur-Yvette, France) and protease inhibitor cocktail (Roche, Newburyport, MA, USA) at 4 °C. Supernatants were collected for further total protein (Pierce BCA proteins Assay kit, Life Technologies), cytokine, and chemokine measurements. The levels of IL-17 cytokine and CCL17 and CXCL1 chemokines were assessed using commercial ELISA kits (detection limits of 15.6 pg/mL for IL-17 and CXCL1 and of 31.2 pg/mL for CCL17), in accordance with the instructions provided by the manufacturers (R&D Systems, Minneapolis, MN, USA). Data are expressed as pg/mg of total proteins.

### 4.7. RNA Isolation and Quantitative RT-PCR

Lung RNA extracts were prepared from the top lobe of the right lung; they were collected and then immersed in RNA*later^®^* (Invitrogen, ThermoFisher Scientific, Waltham, MA, USA) immediately after collection. Lungs were kept in RNA*later^®^* at 4 °C for at least 24 h. After mechanical dissociation (Precellys), RNA was extracted with a nucleospin RNA mini kit (Macherey-Nagel, Hoerdt, France) according to the manufacturer’s instructions. The extracted RNA was reverse-transcribed with the High-Capacity cDNA Archive kit (Applied Biosystems, Foster City, CA, USA) according to the manufacturer’s instructions. Real-time RT-qPCR was performed using StepOne (Applied Biosystems, ThermoFisher Scientific) or QuanStudio 12K Flex Real-Time PCR System (Thermofisher Scientific). Primers (Integrated DNA technologies, Leuven, Belgium) are described in Table 1 and Table 2. Once the PCR amplification was completed, a fusion curve analysis was performed, confirming the presence of a single amplicon. The quantitative RT-PCR cycling was as follows: 1 cycle at 95 °C for 3 min followed by 45 cycles at 95 °C for 5 s and 60 °C for 30 s. Glyceraldehyde-3-Phosphate Dehydrogenase (*Gapdh*) and Hypoxanthine Guanine Phosphoribosyltransferase (*Hprt*) are used as the reference genes in Figure 4 and Figure S8A, respectively. Relative mRNA levels (2^−∆∆Ct^) were determined by comparing the PCR cycle thresholds (Ct) for the gene of interest and the reference gene (∆Ct) and ∆Ct values for the treated and control groups (∆∆Ct).

### 4.8. Histology on Lung Tissue Sections

The left lung was fixed in Antigenfix (Diapath, Microm Microtech, France) for 4 h at room temperature and dehydrated by consecutive baths in increasing concentrations of alcohol (30–100%) and Diasolv (Microm Microtech). After paraffin inclusion, paraffin-embedded lungs were sliced (5 µm) with a Microtom (Microm HM355S Thermoscientific). Sections were stained using periodic acid–Schiff (Microm Microtech) for mucus visualization. Images were acquired on an AxioPlan2 (Zeiss, Oberkochen, Germany) light microscope with Zen Pro software 3.5. The acquired histology photos were analyzed in NDP view 2.0 software (Hamamatsu Photonics K.K., Shizuoka Pref., Japan Japan) using a semi-quantitative severity score (0–5) for inflammatory cell infiltration [55].

### 4.9. Flow Cytometry Analysis of Lung Cells

Mice were anesthetized with isoflurane and received intravenous injection of anti-CD45-PeCy7 antibody (4 µg/mouse, clone 30-F11, Biolegend). Five minutes later, pulmonary blood circulation was washed with 10 mL PBS, and the lungs were collected as previously described [42]. Lung cells were isolated by digesting the tissue in a solution of type IV collagenase (Gibco, ThermoFisher Scientific)-DNase I (Roche) solution at 37 °C for 1 h. The cells were then centrifuged in 30% Percoll solution (GE Healthcare) and counted. Isolated lung cells were stained with the Zombie NIR™ Fixable Viability Kit (Biolegend, San Diego, CA, USA) and FcBlock (ebioscience, Thermofisher scientific) for 20 min at room temperature, then with specific antibodies for 30 min at 4 °C (PerCP anti-mouse CD45 (I3/2.3), FITC anti-mouse CD3 (17A2), BV421 anti-mouse NK1.1 (PK136), PeCy5 anti-mouse CD69 (H1.2F3), and AF647 anti-mouse NKp46 (29A1.4), all from Biolegend). Cells were fixed for 20 min in paraformaldehyde 1% and read on the flow cytometer LSR fortessa (Beckton Dickinson Biosciences, Franklin Lakes, NJ, USA). Analysis was performed using FlowJo software (v10.10). NK cells were identified as CD3^−^NK1.1^+^NKp46^+^ lymphoid cells. Marginated intravascular NK cells were CD45-PeCy7^+^ CD45-PerCP^+^, whereas parenchymal cells were CD45-PeCy7^−^ CD45-PerCP^+^. The gating strategy is given Figure S10.

### 4.10. NK Cell Transfer

C57BL/6 donor mice received FSL-1 by intranasal administration as shown in Figure 7A. Lung cells were isolated as previously described (Section 4.9) and stained with the Zombie Viability Kit and antibodies for CD3, NK1.1, and NKp46. CD3^−^NK1.1^+^NKp46^+^Zombie^−^ NK cells were sorted with ARIA II (BD Biosciences). NK cell purity was above 95%. One million NK cells from FSL-1-treated mice were intravenously injected into recipient OVA-sensitized mice immediately after purification. Sensitized or control recipient mice were analyzed 96 h after the last intranasal challenge with OVA or NaCl (protocol diagram in Figure 7A).

### 4.11. Statistical Analysis

Statistical analysis was performed using GraphPad Prism software 10.2.3. The Shapiro–Wilk test for normality was performed, and a one-way ANOVA was subsequently performed when data followed a normal distribution, followed by Tukey’s multiple comparisons test. Values were then expressed as mean ± standard deviation (SD). Otherwise, a two-way ANOVA or the Kruskal–Wallis test were performed, followed by Tukey’s or Dunn’s multiple comparisons post-test. The significance level was set at *p* < 0.05.

## Figures and Tables

**Table 1:** Primer sequences used for the quantitative PCR in Figure 3.

Target	Forward Primers	Reverse Primers
il-5	5′-AACCCTGAAGTTTCAGGACTCGCCTT-3′	5′-TCTTCAGCGCTGGCCTTCAGCAA-3′
il-10	5′-GGTTGCCAAGCCTTATCGGA-3′	5′-ACCTGCTCCACTGCCTTGCT-3′
il-13	5′-GGGTGACTGCAGTCCTGGCT-3′	5′-GTTGCTCAGCTCCTCAATAAGC-3′
il-17	5′-TCCAGAAGGCCCTCAGACTA-3′	5′-TGAGCTTCCCAGATCACAGA-3′
ifn-γ	5′-TCAAGTGGCATAGATGTGGAAGAA-3′	5′-TGGCTCTGCAGGATTTTCATG-3′
Gapdh	5′-TGTCCGTCGTGGATCTGAC-3′	5′-CCTGCTTCACCACCTTCTTG-3′

**Table 2:** Prime time assays used for the quantitative PCR in Figure S9A.

Target	Catalog Reference	Target	Catalog Reference
ifn-γ	Mm.PT.58.41769240	il-17	Mm.PT.58.6531092
il-5	Mm.PT.58.41498972	muc5ac	Mm.PT.58.42279692
il-10	Mm.PT.58.13531087	muc5b	Mm.PT.58.30457752
il-13	Mm.PT.58.31366752	Hprt	Mm.PT.39a.22214828

## Data Availability

Data are available upon reasonable request from the corresponding author.

## Supplementary Materials

The following supporting information can be downloaded at https://www.mdpi.com/article/10.3390/ijms25179606/s1.
