# Supplementary material for: Pulmonary Administration of TLR2/6 Agonist after Allergic Sensitization Inhibits Airway Hyper-Responsiveness and Recruits Natural Killer Cells in Lung Parenchyma
Source: Int J Mol Sci. 2024 Sep 4;25(17):9606. doi: 10.3390/ijms25179606 (PMC11394962; doi:10.3390/ijms25179606)
Supplement: Supplementary file 1 [file ijms-25-09606-s001.zip › ijms-3146013-supplementary-english.pdf]

Supplemental Data

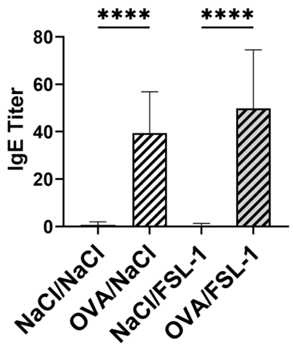

Figure S1. Administration of FSL-1 in the lungs does not modify serum OVA-specific IgE in OVA-sensitized female C57BL/6 mice. Results are expressed as IgE titers related to pooled homemade standards. n=10 NaCl/NaCl, n=10 OVA/NaCl, n=6 NaCl/FSL-1, n=8 OVA/FSL-1. ****p<0.0001 (Shapiro–Wilk test for normality and one-way ANOVA followed by Tukey's multiple comparisons test).

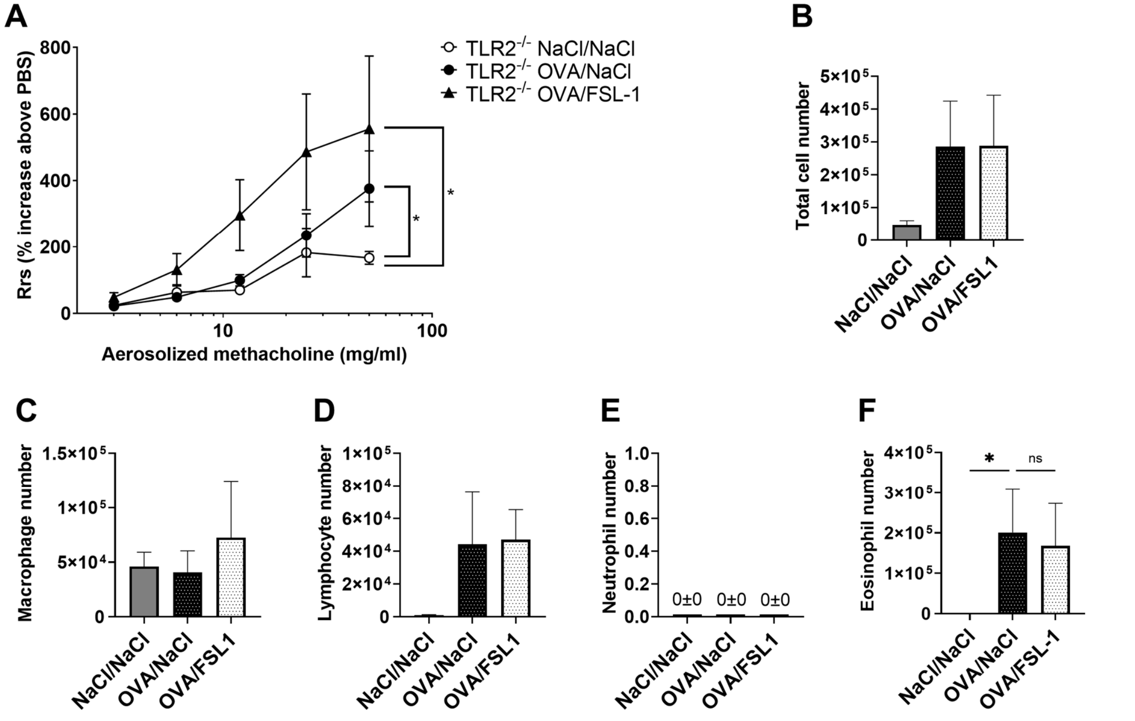

Figure S2. FSL-1 does not modify airway hyper-responsiveness and bronchoalveolar lavage eosinophilia in OVA-sensitized female TLR2$^{-/-}$ mice. A: Results are expressed as percentage of Rrs increase for each methacholine dose in comparison to PBS. n=5 NaCl/NaCl, n=7 OVA/NaCl, n=7 OVA/FSL-1. Statistical significance is shown for the highest methacholine dose: *p<0.05 (two-way ANOVA followed by Tukey's multiple comparisons test). B-F: Results are expressed as number of cells. n=5 NaCl/NaCl, n=7 OVA/NaCl, n=7 OVA/FSL-1. * p<0.05, ns = non-significant (p = 0.8724) (Shapiro–Wilk test for normality and one-way ANOVA followed by Tukey's multiple comparisons test).

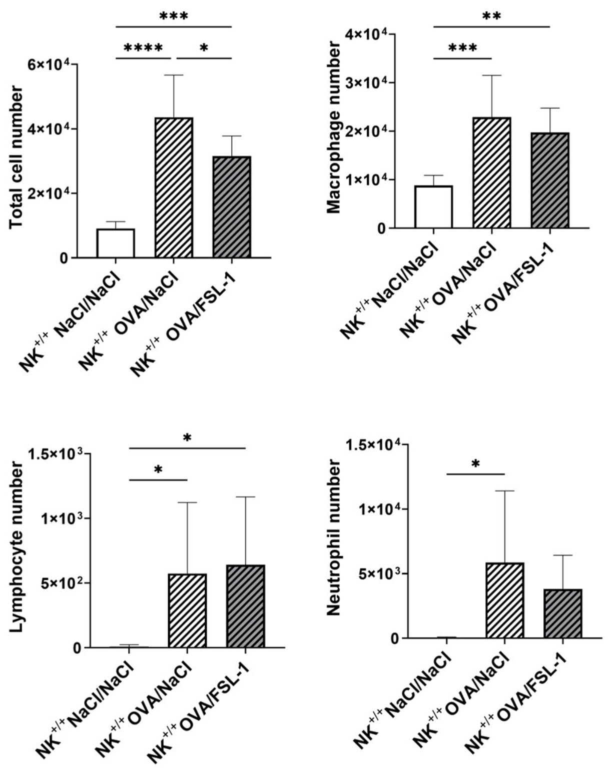

Figure S3. FSL-1's effects on bronchoalveolar lavage cellularity in female littermate NK$^{+/+}$ mice. Bronchoalveolar lavage cells corresponding to mice of figure 9A and B. n=9 NK$^{+/+}$ NaCl/NaCl mice, n=9 NK$^{+/+}$ OVA/NaCl mice, n=8 NK$^{+/+}$ OVA/FSL-1 mice. * $p<0.05$, **$p<0.01$, ***$p<0.001$, ****$p<0.0001$ (Shapiro–Wilk test for normality and one-way ANOVA followed by Tukey's multiple comparisons test).

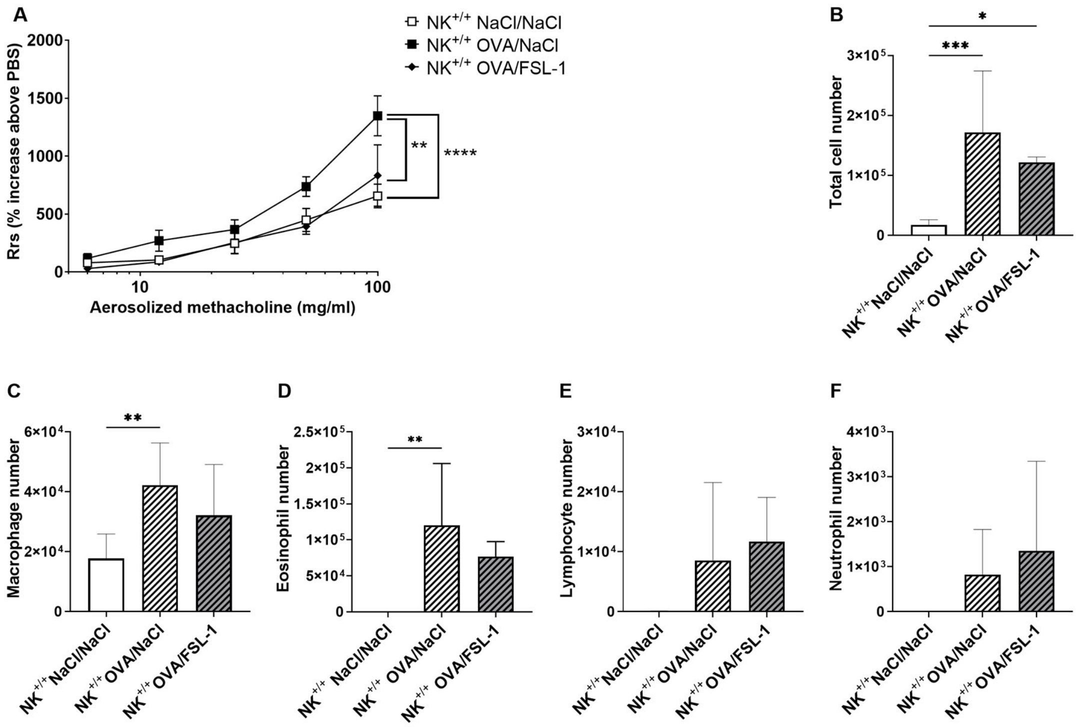

Figure S4. FSL-1 decreases airway hyper-responsiveness in OVA-sensitized male mice. $NK^{+/+}$ male littermates for $NK^{-/-}$ male mice were analyzed for their response to FSL-1 administration in the lungs after OVA sensitization. A: Lung resistance expressed as percentage of Rrs increase for each methacholine dose in comparison to PBS. n=7 NaCl/NaCl, n=10 OVA/NaCl, n=5 OVA/FSL-1. Statistical significances are shown for the highest methacholine dose: **p<0.01, ****p<0.0001 (two-way ANOVA followed by Tukey's multiple comparisons test). B-F: Cellularity in bronchoalveolar lavage. n=7 NaCl/NaCl, n=9 OVA/NaCl, n=5 OVA/FSL-1. * p<0.05, ** p<0.01, ***p<0.001 (Shapiro–Wilk test for normality and one-way ANOVA followed by Tukey's multiple comparisons test).

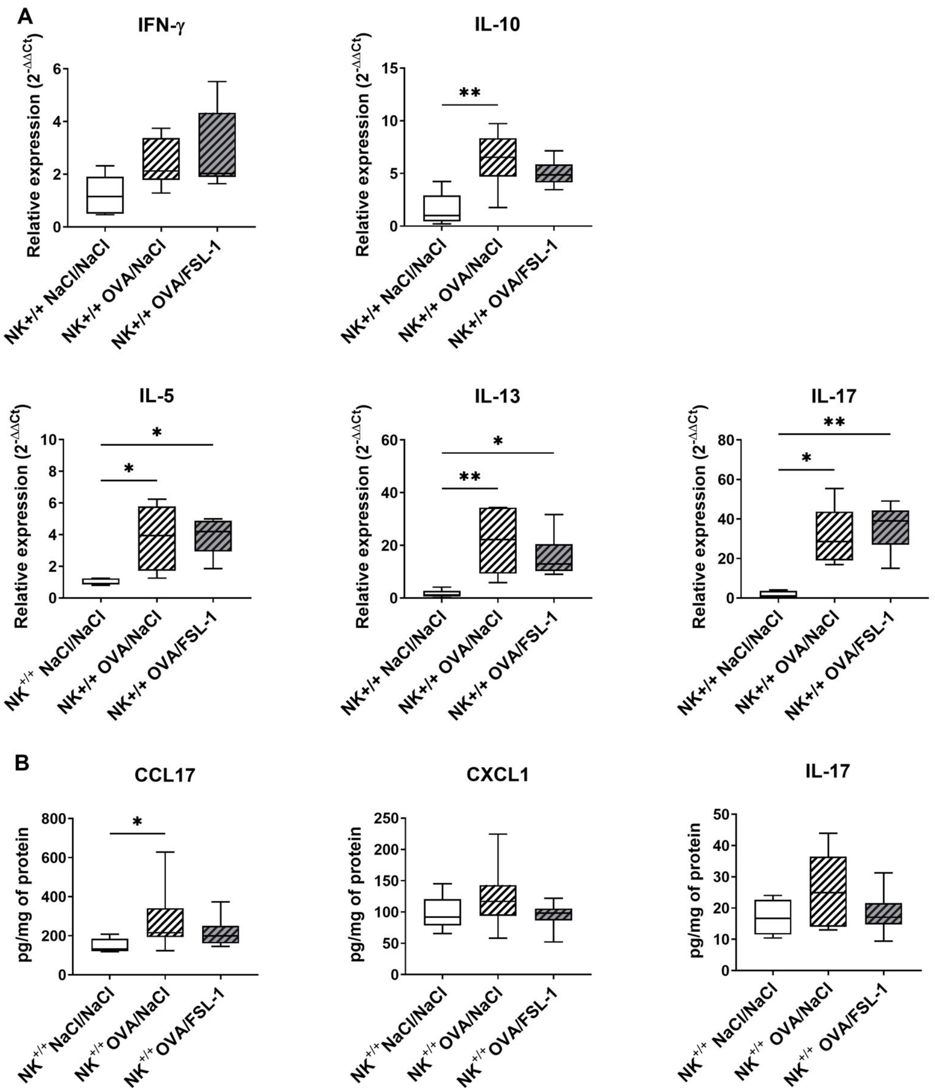

Figure S5. FSL-1's effect on cytokine and chemokine expression in the lungs of female littermate NK$^{+/+}$ mice. A: mRNA expression in lung extracts by qRT-PCR. Relative expression was determined as $2^{-\Delta\Delta Ct}$. HPRT was used as housekeeping gene. n=5 NaCl/NaCl, n=6 OVA/NaCl, n=7 OVA/FSL-1. * p<0.05, ** p<0.01 (Kruskal–Wallis test followed by Dunn's multiple comparisons post-test). B: Cytokine levels in lungs expressed as pg/mg of total lung proteins. n=5 NaCl/NaCl, n=10 OVA/NaCl, n=10 OVA/FSL-1. * p<0.05 (Kruskal–Wallis test followed by Dunn's multiple comparisons post-test).

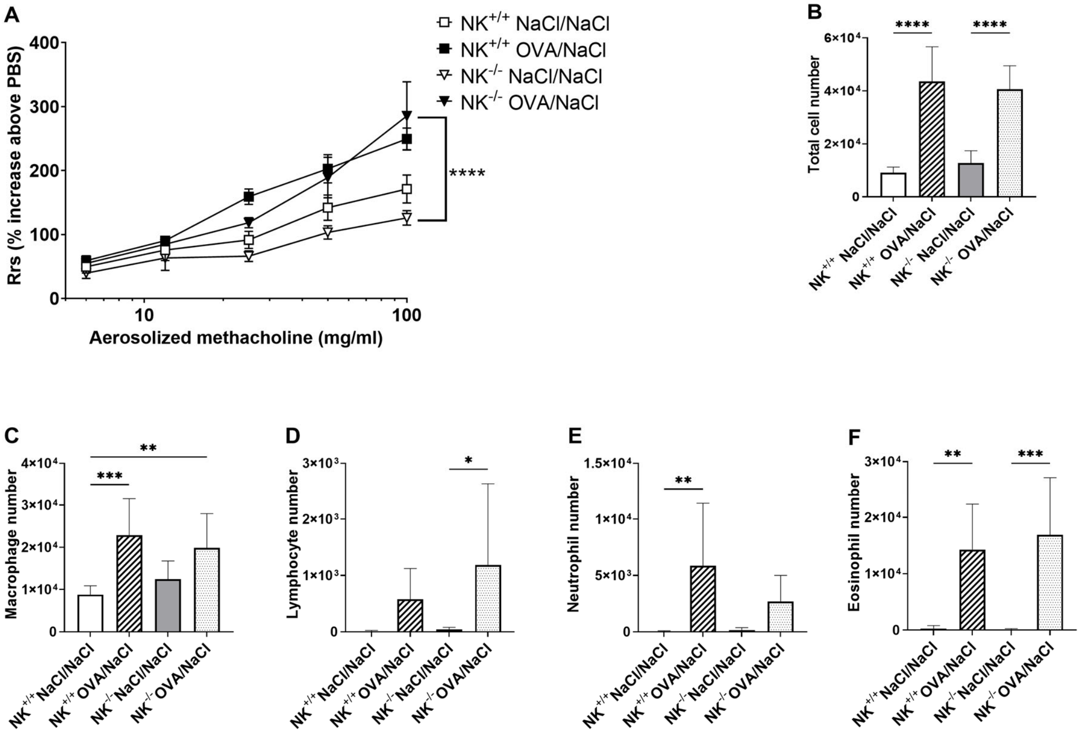

Figure S6. NK cells are not essential for OVA-induced airway hyper-responsiveness and bronchoalveolar lavage eosinophilia. Airway resistance (A) and bronchoalveolar lavage cell numbers (B-F) were measured in NK$^{-/-}$ and their littermate NK$^{+/+}$ female mice treated as mentioned in figure 1. NK$^{+/+}$ mice: n=9 NaCl/NaCl, n=9 OVA/NaCl. NK$^{-/-}$ mice: n=6 NaCl/NaCl, n=12 OVA/NaCl. * $p<0.05$, **$p<0.01$, ***$p<0.001$, ****$p<0.0001$. A: Statistical significance is shown for the highest methacholine dose (two-way ANOVA followed by Tukey's multiple comparisons test). B: Shapiro–Wilk test for normality and one-way ANOVA followed by Tukey's multiple comparisons test.

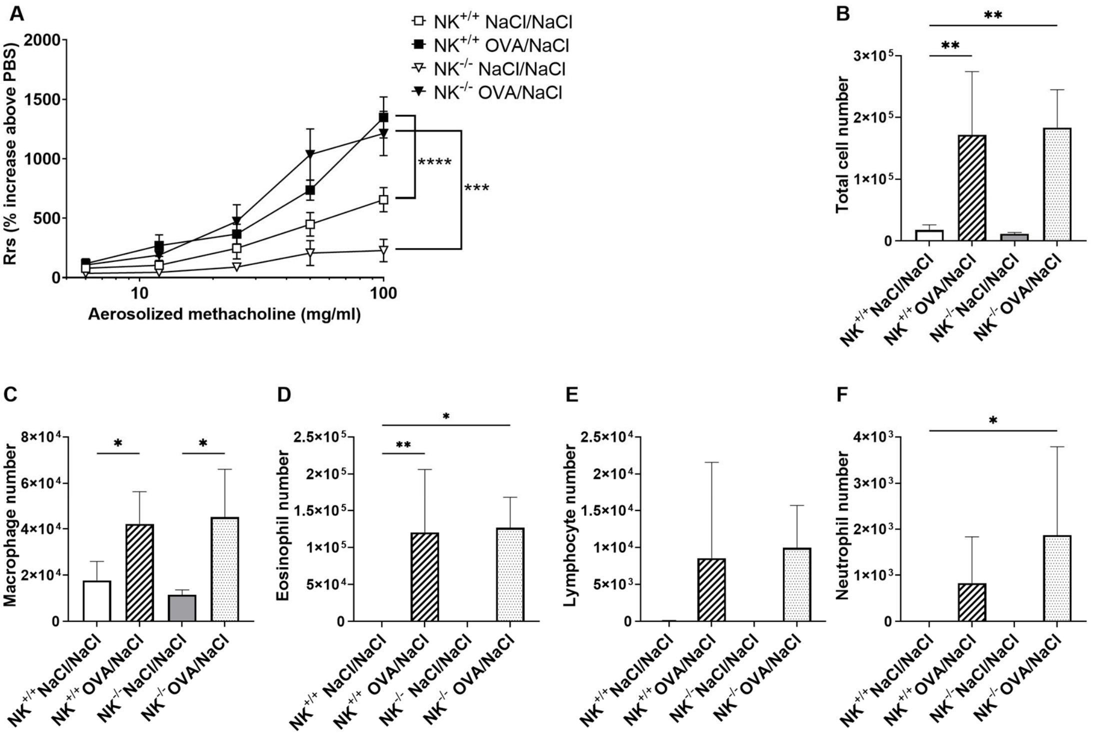

Figure S7. NK cell depletion does not affect airway hyper-responsiveness and cellularity in bronchoalveolar lavage in OVA-sensitized male mice. $NK^{-/-}$ male mice were analyzed for their response to OVA sensitization in comparison to littermate $NK^{+/+}$ males. A: Lung resistance expressed as percentage of Rrs increase for each methacholine dose in comparison to PBS. n=7 $NK^{+/+}$ NaCl/NaCl, n=10 $NK^{+/+}$ OVA/NaCl, n=2 $NK^{-/-}$ NaCl/NaCl, n=4 $NK^{-/-}$ OVA/NaCl. Statistical significance is shown for the highest methacholine dose. ***$p<0.001$, ****$p<0.0001$ (two-way ANOVA followed by Tukey's multiple comparisons test). B-F: Cellularity in bronchoalveolar lavage. n=7 $NK^{+/+}$ NaCl/NaCl, n=10 $NK^{+/+}$ OVA/NaCl, n=2 $NK^{-/-}$ NaCl/NaCl, n=4 $NK^{-/-}$ OVA/NaCl. * $p<0.05$, ** $p<0.01$ (Shapiro–Wilk test for normality and one-way ANOVA followed by Tukey's multiple comparisons test).

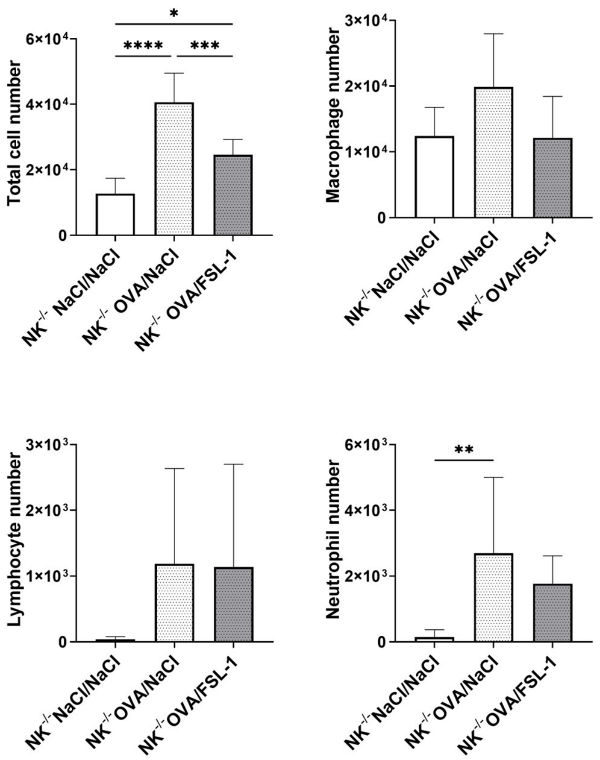

Figure S8. FSL-1's effects on bronchoalveolar lavage cellularity in OVA-sensitized female NK$^{-/-}$ mice. Bronchoalveolar lavage cells corresponding to mice of figure 8B. n=6 NK$^{-/-}$ NaCl/NaCl mice, n=12 NK$^{-/-}$ OVA/NaCl mice, n=7 NK$^{-/-}$ OVA/FSL-1 mice. * p<0.05, **p<0.01, ***p<0.001, ****p<0.0001 (Shapiro–Wilk test for normality and one-way ANOVA followed by Tukey's multiple comparisons test).

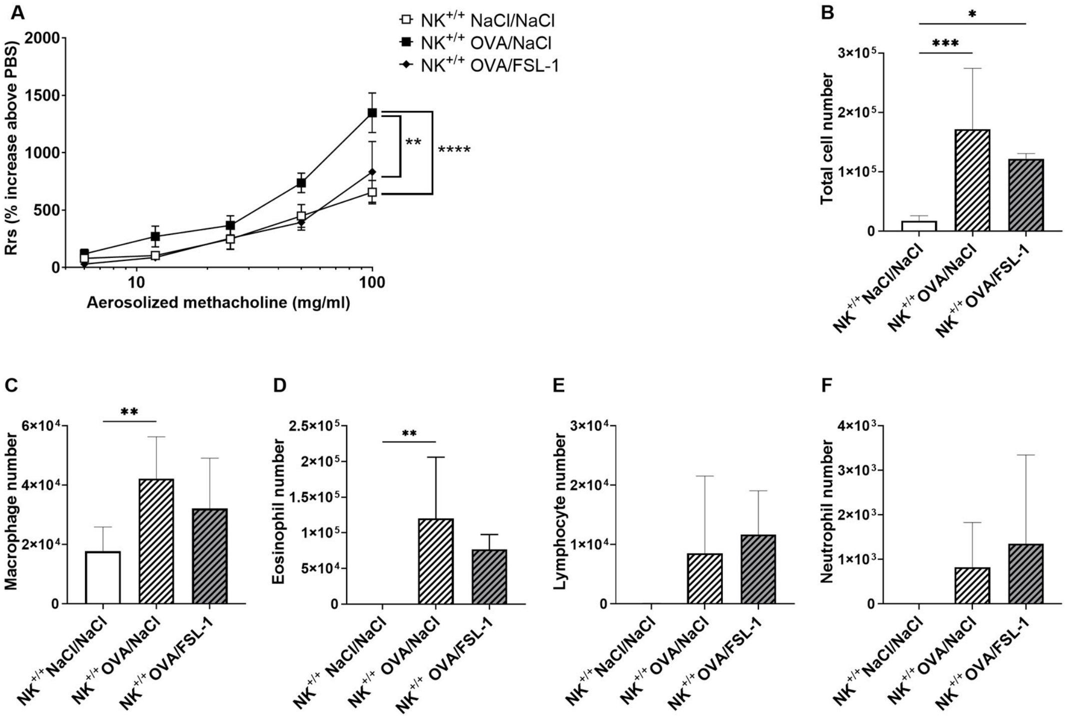

Figure S9. FSL-1 decreases airway hyper-responsiveness in OVA-sensitized male mice. NK$^{+/+}$ male littermates for NK$^{-/-}$ male mice were analyzed for their response to FSL-1 administration in the lungs after OVA sensitization. A: Lung resistance expressed as percentage of Rrs increase for each methacholine dose in comparison to PBS. n=7 NaCl/NaCl, n=10 OVA/NaCl, n=5 OVA/FSL-1. Statistical significance is shown for the highest methacholine dose. **p<0.01, ****p<0.0001 (two-way ANOVA followed by Tukey's multiple comparisons test). B-F: Cellularity in bronchoalveolar lavage. n=7 NaCl/NaCl, n=9 OVA/NaCl, n=5 OVA/FSL-1. * p<0.05, ** p<0.01, ***p<0.001 (Shapiro–Wilk test for normality and one-way ANOVA followed by Tukey's multiple comparisons test).

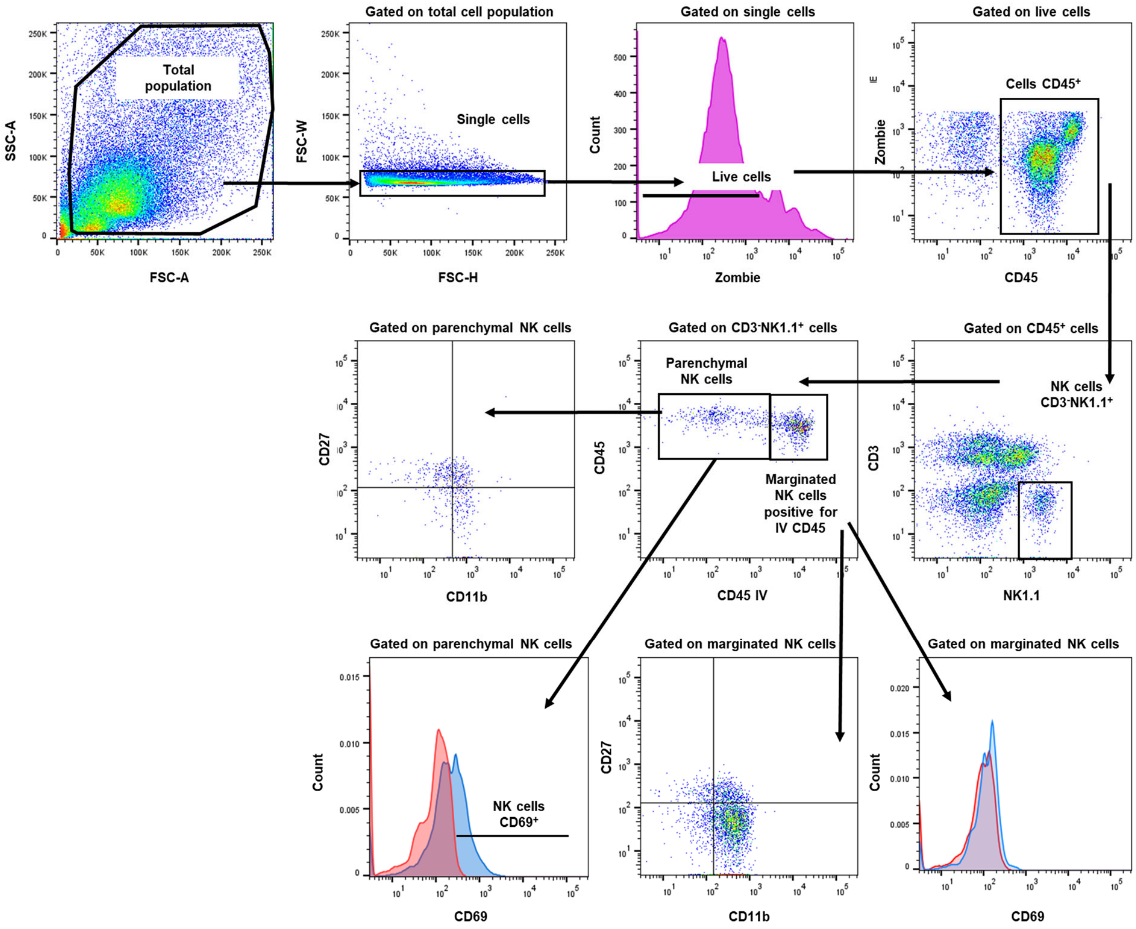

Figure S10: Flow cytometry gating strategy. We gated for single and live cells within the lung cell populations. NK cells were identified as lymphoid cells expressing CD45 and NK1.1 but lacking CD3 expression. Marginated and parenchymal NK cells were defined by administering an intravenous injection (IV) of an anti-CD45 antibody prior to ex vivo staining. CD45 IV^{+}CD45^{+}NK1.1^{+}CD3^{-} cells were identified as marginated NK cells, and CD45 IV^{-}CD45^{+}NK1.1^{+}CD3^{-} cells as parenchymal NK cells. To define the maturation and activation of NK cells, the surface expression of CD69, CD11b, and CD27 on both populations was used.
